# Attitudes of Patients and Health Professionals Regarding Screening Algorithms: Qualitative Study

**DOI:** 10.2196/17971

**Published:** 2021-08-09

**Authors:** Christina Oxholm, Anne-Marie Soendergaard Christensen, Regina Christiansen, Uffe Kock Wiil, Anette Søgaard Nielsen

**Affiliations:** 1 Department for the Study of Culture University of Southern Denmark Odense Denmark; 2 The Maersk Mc-Kinney Moller Institute University of Southern Denmark Odense Denmark; 3 Research Unit of Clinical Alcohol Research Department of Clinical Research University of Southern Denmark Odense Denmark

**Keywords:** screening, algorithms, alcohol, qualitative study, attitudes, opinions, patients, health professionals

## Abstract

**Background:**

As a preamble to an attempt to develop a tool that can aid health professionals at hospitals in identifying whether the patient may have an alcohol abuse problem, this study investigates opinions and attitudes among both health professionals and patients about using patient data from electronic health records (EHRs) in an algorithm screening for alcohol problems.

**Objective:**

The aim of this study was to investigate the attitudes and opinions of patients and health professionals at hospitals regarding the use of previously collected data in developing and implementing an algorithmic helping tool in EHR for screening inexpedient alcohol habits; in addition, the study aims to analyze how patients would feel about *asking* and *being asked* about alcohol by staff, based on a notification in the EHR from such a tool.

**Methods:**

Using semistructured interviews, we interviewed 9 health professionals and 5 patients to explore their opinions and attitudes about an algorithm-based helping tool and about asking and being asked about alcohol usage when being given a reminder from this type of tool. The data were analyzed using an ad hoc method consistent with a close reading and meaning condensing.

**Results:**

The health professionals were both positive and negative about a helping tool grounded in algorithms. They were optimistic about the potential of such a tool to save some time by providing a quick overview if it was easy to use but, on the negative side, noted that this type of helping tool might take away the professionals’ instinct. The patients were overall positive about the helping tool, stating that they would find this tool beneficial for preventive care. Some of the patients expressed concerns that the information provided by the tool could be misused.

**Conclusions:**

When developing and implementing an algorithmic helping tool, the following aspects should be considered: (1) making the helping tool as transparent in its recommendations as possible, avoiding black boxing, and ensuring room for professional discretion in clinical decision making; and (2) including and taking into account the attitudes and opinions of patients and health professionals in the design and development process of such an algorithmic helping tool.

## Introduction

### Background

The more specialized hospital treatments and hospital departments become, the more challenging it will be to maintain an overview of all the information collected in the hospitals’ electronic health records (EHRs). It has been suggested that a software algorithm may be a reliable strategy for automatic screening of EHRs [1]. Software using data mining, machine learning, and natural language processing may not only prove to be useful for overseeing the vast amount of data in the EHRs but using software to generate automatic messages to health professionals for decision making may also remove barriers when it comes to talking to their patients about sensitive topics such as alcohol.

### Hospitalized Interventions Aimed at Reducing Harmful Alcohol Intake

There is an overwhelming body of evidence proving the negative influence of the substantial use of alcohol in the areas of both public health and health economics. Alcohol is a significant risk factor for bad health and premature death, and alcohol use disorder is responsible for considerable physical morbidity and injuries [2]. Alcohol is leading to, or complicating, at least 60 diagnoses, and excessive drinking in Denmark alone is related to 28,000 hospital admissions, 10,000 emergency room visits, and an additional annual cost of health services of DKK 947 million (US $151.05 million) [3].

In studies on recovery from alcohol problems, health problems and hospital admissions are among the most cited predictors of recovery [4]. Health problems and hospital admissions may open a window for changing alcohol consumption if this opportunity is exploited. In other words, general hospitals may be in a good position to identify individuals with alcohol issues. Hence, the screening, brief intervention, and referral to specialized treatment (SBIRT) approach for alcohol use disorder during hospitalization is considered suitable to address and lower the alcohol intake of patients [5].

The SBIRT approach aims to identify patients with high alcohol intake (the screening component). When identified, the next step is to increase the patients’ awareness of their alcohol intake and increase their motivation to lower it (the brief intervention component), which is most often based on the principles of motivational interviewing [6]. The final part of the intervention sends the patients who need treatment for alcohol use disorder to specialized treatment (the referral to treatment component). A series of projects have tried to implement the SBIRT approach, but it has proved indeed very challenging [7,8].

In particular, it seems that health professionals are reluctant to both screen for risky alcohol use among patients due to the time spent on the procedures, and address drinking issues when the patients screen positive [9,10]. Health professionals express, however, that they are willing to talk about alcohol with their patients if they have a reason for doing so [10,11]. One difficult barrier seems to be that the excessive alcohol intake, even daily, is hard to observe and detect because large alcohol intake is often invisible. Therefore, health professionals may be afraid of insulting nondrinkers if they screen for excessive alcohol use systematically. Hence, health professionals’ discomfort and avoidance of the topic have led to alcohol problems being ignored [12,13].

### Helping Tools for Health Professionals Based on Data From EHRs

Predictive models based on EHR data may be a way to help health professionals at hospitals to identify patients with alcohol use disorder. Data mining and machine learning techniques have been used extensively for predictive models and clinical decision support. Indeed, Escobar et al [14,15] aimed to predict the occurrence of an adverse event to avoid hospitalized patients being transferred to the intensive care unit. Hackmann et al [16] developed a clinical warning system that simultaneously reduces the risk of false positives while ensuring that the right patients are administered into the monitoring program. Mishra et al [17] analyzed discharge summaries to identify diabetes, protocol compliance, and high-risk factors; this was also done using simple concept extraction methods. So far, an algorithm-based helping tool that screens EHR data to inform hospital staff that the patient might have a complicating use of alcohol that should be addressed in order to improve the prognosis of the patient has not been developed [18].

However, using machine learning techniques, it seems possible to develop an algorithm that screens data already stored in patient case notes and that can be the backbone of a clinical decision tool for identifying possible harmful alcohol use [19]. If successful, the clinical decision tool would give a message to health professionals if the patients screen positive for harmful use. The tentative models for developing reliable algorithms central to such tool are promising [19]. However, before developing predictive models and algorithms that are ready to be implemented in clinical practice, we need to know if a clinical decision support tool, which can scan data already stored in patient case notes in EHRs and informing health professionals about indications of harmful use of alcohol, is considered acceptable by patients and health professionals. The risk is that such a tool will be perceived as unethical and as “big brother is watching you.”

There are a several reports mapping the ethical issues involved in the development and use of clinical decision support tools on theoretical levels [20], and various studies on how to achieve user acceptance of such tools [21]. However, we lack knowledge on the attitudes and ethical considerations of patients and health care professionals in relation to such tools. Thus, this study will investigate the attitudes and ethical considerations among both health professionals at hospitals and patients toward using patient data already stored in the EHR, to develop an algorithm and a subsequent helping tool to inform staff that harmful alcohol use may be a complicating factor for the patient in question.

## Methods

### Recruitment Process/Strategy

The participants were primarily recruited at Odense University Hospital, Denmark, in the Department of Neurology, the Department of Orthopedics and Traumatology, and the Department of Gastroenterology. Our overall goal was to interview 2-3 health professionals and 2-3 patients in each department. Because of the short and varying number of admissions and an unpredictable work environment, we were unable to ensure a specific number and variation of participants in advance.

Unfortunately, there were not enough patients on the days of the interviews who were fit enough or willing to be interviewed. Because we did not get enough diversity of opinions with the admitted patients, we decided to expand our study to include nonadmitted potential patients to achieve thematic saturation. The rationale for including nonadmitted potential patients was that everyone is a *potential* patient and has been seen by a doctor at some point in their lives and can therefore relate to and have opinions about being asked about their alcohol use by a health professional. Furthermore, potential patients often have a greater surplus of mental resources to consider the questions in the interview than ill, hospitalized patients. Therefore, 2 participants in the patient group were recruited through an open call via the University of Southern Denmark’s network.

### Ethics, Consent, and Permission

Because the project was solely based on voluntary interviews and did not involve any biological material, the Regional Committees on Health Research Ethics for Southern Denmark ruled that there was no need to apply for permission to conduct the interviews.

The heads of each medical department were contacted via email and given thorough information about the project beforehand. Together, we scheduled a date for conducting the interviews. Three separate dates were arranged with each of the medical departments.

To ensure that we did not interview patients who were either not fit enough or unable to give informed consent, we consulted with the health professionals. The health professionals selected which patients would be physically and mentally capable of participating. This gave us ethical assurance that the patients who were interviewed would not suffer in terms of well-being and could, in fact, provide informed consent. The patients were first approached by one of the health professionals who gave them a basic outline of the project and asked them if they would like to learn more and, potentially, participate. This was to ensure that the patients did not feel uncomfortable when being approached by a stranger. Besides, making the health professional establish first contact made our project more trustworthy and credible. If the patient wanted to hear more about the project, the interviewer (CO) presented herself and the project in detail.

Before starting the interviews, we obtained written consent from all the participants. The consent ensured the participants their confidentiality and anonymity. The participants were also informed that they would be able to withdraw their consent at any time in the process.

### Data Collection

This study makes use of a hermeneutic-phenomenological approach. Because of the study’s clear aim and limited time available for both admitted patients and health professionals, we decided that semistructured interviews would be the best way to approach our research question. We developed the interview guide in a relatively open manner to avoid influencing the participants’ attitudes with specific words or phrases. The interview guide was adjusted by conducting a series of test interviews to ensure that the questions were understandable and that the interview was structured in the right way.

Because of the study’s explorative focus on patient and health professional’s attitudes toward, and opinions on, use of technology in health care rather than technology compliance, we did not base this study on a technology acceptance framework.

In total, 14 participants were interviewed (9 health professionals and 5 patients). The health professionals were selected by the head of each department, depending on their availability. Table 1 provides an overview of the group of health professionals interviewed in the study. The interviews of the health professionals were conducted in a small, quiet meeting room in each department.

A total of 5 patients were interviewed for this study, 3 of whom were patients admitted at the Odense University Hospital. The remaining 2 patients were recruited through the open call. The interviews with the admitted patients took place in their respective rooms in the hospital. The nonadmitted patient interviews were held at the University of Southern Denmark in a private office. Table 2 presents the gender of the patients interviewed.

**Table 1:** Health professionals: an overview.

Gender	Doctors, n	Nurses, n	Social and health service assistant, n
Female	2	4	2
Male	1	—	—

**Table 2:** Gender of the patients.

Gender	Patients, n
Female	3
Male	2

All interviews had a maximum duration of 30 minutes and were audio recorded. The interviews focused on patients’ and health professionals’ views on addressing the patients’ alcohol use during hospitalization and the patients’ and health professionals’ views on developing automatic screening tools based on algorithms that can screen EHRs for signs of problematic alcohol use. All interviews were conducted by the same person (CO) who used a semistructured interview guide. CO does not have a health-related educational background. Thus, she did not have any specific ideas about good and bad practices in health care and was not familiar with the health profession’s norms and rules beforehand. This allowed her to be open toward participants and perform the interviews without any prejudice.

### Data Analysis

The audio files were transcribed in MSWord and analyzed ad hoc. The analysis primarily involved a process of close reading of the printed transcriptions and meaning condensing and color coding by hand. This process did not include any computer-assisted qualitative software. The analysis was primarily carried out by CO and sent back and forth to A-MC, RC, and AN, who then evaluated and gave their feedback on the analysis independent of each other. A-MC, RC, and AN also had the full transcriptions and, therefore, could make qualified comments on something that might have been missed or that needed further exploration. The data were analyzed for both the patients’ and health professionals’ views on addressing alcohol use during hospitalization, and for the patients’ and health professionals’ views on developing an algorithm and screening existing EHRs. The patients’ and health professionals’ views on addressing alcohol abuse are presented elsewhere [11]. This paper focuses solely on the attitude and ethics regarding automatic screening of EHRs for signs of alcohol abuse.

## Results

### Overview

To investigate the health professionals’ and patients’ opinions on and attitudes toward an algorithm-based helping tool for screening alcohol habits, the questions in the interview contained 2 different perspectives. First, the interview questions asked about the respondent’s general views on an algorithm-based helping tool and their opinions about *asking* and *being asked*, based on a reminder from the same helping tool.

### Health Professionals

#### Interview Outcomes

To unfold health professionals’ opinions on an algorithm-based helping tool, they were asked about the general use of information technology (IT) in health care (mainly EHR and other IT systems regarding registration and documentation). This was done to test whether a possible negative opinion about the general use of IT in health care was affecting their opinion regarding a specific helping tool. Interrogating the health professionals’ opinions and attitudes about *asking* about alcohol, based on a reminder from the helping tool, was a way to make them consider the helping tool from a more practical perspective. This would uncover any inconsistencies in their answers.

#### Opinions on the General Use of IT in Health Care

Regarding the question about their general opinions on the use of IT in health care, 8 out of the 9 respondents had both positive and negative opinions, with the remaining 1 being entirely positive about the general use of IT in health care. On the positive side were, for example, that the EHRs’ availability saves valuable time compared with the old paper journals, and that EHRs provide a quick and useful overview of the patients. Even though none of the health professionals explicitly expressed this claim, the fact that all 9 of them mentioned time saving as a positive aspect of IT in health care serves as a strong indicator that time may be limited, and therefore, a valuable resource in health care.

On the negative side, a recurring theme among the health professionals was that the documentation requirements following EHRs were considered time-consuming. Some added that it sometimes was experienced as meaningless, for example, because it doubled documentation and took valuable time away from the patients.

One health professional (HP-2) expressed this last point by saying that having to withdraw from the patients to spend a large amount of time in front of a computer was not why she became a health professional. At the same time, she also expressed a view on IT as having many advantages; for example, it can save a lot of time when reading up on patients. Another health professional (HP-7) who also had mixed opinions about IT pointed to some specific challenges: (1) that the restrictions on access to information are a limitation because this can be a hindrance for some health professionals when accessing valuable information; (2) that some of the IT systems do not work together, which results in professionals having to document the same thing in 2 different systems; and (3) that the high documentation requirements produce a vast amount of data, which can be difficult to navigate through when trying to find specific information. As HP-2, HP-7 also expressed positive views on the use of IT in health care when compared with old paper journals. These 2 examples sum up most of the health professionals’ ambivalence toward the general use of IT in health care: the paradox that IT both saves time and requires a lot of time. Although most of the health professionals shared this ambivalent opinion, no one was exclusively negative about the general use of IT in health care.

#### Opinions on an Algorithm-Based Helping Tool

Six out of the 9 health professionals (HP-1, HP-5, HP-6, HP-7, HP-8, and HP-9) were predominantly positive about the idea of an automatic and algorithm-based helping tool, with 1 (HP-2) being exclusively positive about it. The remaining 2 health professionals (HP-3 and HP-4) were ambivalent. A common denominator among all 9 health professionals in their positive opinions about this type of helping tool was how these opinions almost exactly mirrored their positive opinions about IT in general: the possibility of *saving time* thanks to the automatic sorting of data, getting a *quick overview* of the relevant issues concerning the individual patient, and making sure that they do not forget important tasks by being reminded of them (especially for new health professionals).

Although 6 of the health professionals were predominantly positive and expressed that they would consider the tool helpful in their work, they also expressed that their positive attitudes were conditioned by the following requirements: (1) that the helping tool would be adapted to each department’s field of expertise, (2) that it would not be time-consuming, (3) that there would be sufficient time and resources spent on its implementation, (4) that the helping tool would actually work and be useful, (5) that the tool would be designed in a user-friendly way, and (6) that the system would not be implemented in a top–down manner. The conditions were distributed across the answers of 6 of the respondents. Even though the respondents all had some reservations, they were categorized as being predominantly positive, because they did not object to anything related to the helping tool itself, but rather to some conditions surrounding it.

HP-1, who expressed that she would want the helping tool to be adapted to each department’s field of expertise, elaborated that she would find it very frustrating if such a tool was implemented in a top–down manner without any consideration of how the tool would affect or benefit the ones using it. In her opinion, not all initiatives would be equally relevant for every field of expertise, and if the tool provides some users with irrelevant information, it would be more of a disturbance than a benefit. If the tool would be adapted according to relevance, she would be positively disposed toward this kind of technology. HP-1’s reservation could point to a more general frustration that health professionals experience: not being included in the development and implementation of systems or guidelines that have a substantial influence on how they work.

HP-9, who expressed the condition of a user-friendly design, also mentioned a possible pitfall if the data used for the screenings were inadequate or incomplete. In other words, if the health professionals—for one reason or another—did not document items correctly, sufficiently, or in the right place, then the screenings could potentially produce errors (eg, in the form of faulty recommendations).

Although none of the health professionals were exclusively negative about the idea, 2 (HP-3 and HP-4) were clearly ambivalent in their opinions on the matter and were neither predominantly positive nor predominantly negative. Their reservations about the helping tool were of a more fundamental character than the ones expressed by those with predominantly positive opinions because they touched upon how algorithm-based helping tools could affect the nature of being a health professional.

Even though HP-3 was very positive about this type of helping tool, stating that she would consider such a tool helpful in her work, she also expressed a profound concern that the tool would take away the health professionals’ instinct: “I think that it is fine to make them [the helping tools], but you have to be careful that it does not become a false safety for the health professionals, that they are going to use it blindly without using...the instinct”. She was concerned that the helping tools would make health professionals “lazy” by following their instructions blindly without exercising professional discretion. She had mixed feelings on the matter, because while having this concern, she could also see great potential benefits from having this type of tool.

HP-4 was also very ambivalent about helping tools. On the one hand, she was very positive about algorithms assisting her with medication interactions, but very negative about algorithms assisting her in the diagnostic process. In her opinion, algorithms cannot make complex assessments regarding a patient’s health status. She gave an example of a triage algorithm that evaluates the status of acute patients according to 5 different categories. In her experience, triage algorithms are more of a constraint than help, because they either triage some irrelevant parameter very high or do not catch very serious conditions. According to her, triage algorithms are generally wrong because they cannot evaluate complex medical problems such as a patient’s health status. Therefore, she relies more on her expertise and professional discretion than on these algorithms. This triage algorithm, she said, sometimes makes it difficult for her to be a good clinician because it interferes with her professional judgments: “(...) it can be a problem being...to be an understanding...a good clinician when there is too much that becomes algorithm-based (...)”. But this does not mean that HP-4 was negative about algorithm-based helping tools per se, but rather that she was negative or skeptical about their *usefulness* and *competence* to do certain tasks, for example, assessing a patient’s health status. If a helping tool could help in more “black-and-white” matters (eg, medication interactions), she was very positive about such initiatives.

Most of the health professionals we interviewed had a predominantly positive attitude regarding helping tools based on algorithms, despite most of them being rather ambivalent regarding the *general* use of IT in health care. Nothing suggests that the health professionals’ mixed opinions about IT in general affected their opinions and attitudes regarding the idea of an algorithm-based helping tool.

#### Asking on the Basis of a Reminder

A total of 6 of the 9 respondents were positive about having to ask their patients about alcohol based on a reminder from an algorithm-based helping tool. Most of them stated that they would consider it a help in their work, and some even said that they thought the helping tool would make it *easier* for them to ask patients, because it would give them a sense of having a valid reason or excuse to ask the patients about the patients’ alcohol habits (HP-1, HP-3, and HP-7). These answers indicate that asking about alcohol can be a difficult task for some health professionals. Another reason behind this positive attitude was that the helping tool would ensure that they would remember to ask the patients in the first place.

One of the participants (HP-6) expressed mixed opinions about asking patients based on being reminded by an algorithm. She stated that she would be critical about the reminder and first examine if she agreed that alcohol would be a relevant thing to ask about a specific case. If she were to agree with the algorithm, she would be positive about being reminded. But if this was not the case and she did not find it relevant, she stated that she would ignore the reminder and not ask the patient. This skeptical attitude was also present in her answer regarding whether she would consider the helping tool a help or nuisance in her work. Here, she stated that she would consider the system a help in her work only when she would otherwise have overlooked the relevance of alcohol.

HP-4, who was very negative about algorithm-based helping tools making clinical assessments about patients’ health status, would ignore a reminder asking her to talk to the patients about their alcohol habits. Her reasons behind ignoring the algorithm were similar to the ones she gave when asked about the helping tool in general: She did not think that algorithms can provide clinical assessments, because this is too complex a task for a computer system, which is why she would ignore the reminder and rely on her professional intuition instead of an algorithm.

HP-8 stands out here by not having any problem with asking about alcohol based on a reminder, while also pointing out that the alcohol habits of patients are not of any interest to her. This means that HP-8 would not be likely to ask at all—reminded or not—because of the conviction that this issue is of little relevance in her work. The problem for HP-8 is thus not the reminder of the helping tool per se, but alcohol as a subject. HP-8 might be positive about asking about other cases based on a reminder.

### Patients

#### Interview Outcomes

To investigate their opinions about an algorithm-based helping tool, the patients were also asked about their opinions on the general use of IT in health care (mainly EHRs and other IT systems). This question was asked to uncover if there was a potential negative attitude about the helping tool arising out of a negative or skeptical attitude about the use of IT in health care in general.

To discover the patients’ opinions of the practical use of the helping tool, they were asked several—but related—questions on this matter. These questions included their views on the following: (1) using a helping tool to screen for increased risk or disposition for specific diseases (eg, Alzheimer, cancer, strokes), (2) using a helping tool to screen for signs of alcohol and lifestyle diseases, (3) having their personal EHRs screened for alcohol habits, and, lastly (4) being *asked* about alcohol habits by a health professional, who was reminded to do so by an algorithm-based helping tool. Questions 1 and 2 were asked to test whether it was the *helping tool* or the *use* of the helping tool that they might have an issue with. Questions 3 and 4 were used to uncover whether their general opinion about the helping tool also applied to cases concerning themselves.

#### Opinions on the General Use of IT in Health Care

All patients had a positive attitude about the general use of IT in health care. One of the patients was exclusively positive about the general use of IT, with the remaining 4 being predominantly positive but at the same time expressing some reservations about the potential negative side effects connected to using IT in health care. The concerns raised by the 4 respondents were as follows: (1) that IT could take away valuable time from the patients, (2) that EHRs are more susceptible to abuse than the old paper journals, and (3) that the patients’ access to their own EHRs may be a cause of unnecessary worry. Again, this listing of concerns is not to be perceived as if all the patients raised each of these concerns.

The last concern was raised by P-5, who was interviewed as a patient, but worked as a health professional. Her point was not that patient access to their EHRs is a negative thing, but that it does have the potential to make patients feel anxious if they read the doctors’ notes online and do not understand the medical terms used. This is possibly because the doctors’ notes and test results are often available in the online and patient-accessible EHRs before the patients’ appointment with their doctor. The concern about patient access to EHRs online was raised because she had experienced this sort of dilemma in her personal life and saw what kind of fear it could spark in a patient—sometimes for no reason.

On the positive side regarding the general use of IT in health care, the patients mentioned the following: (1) that using IT is an inevitability and the right step forward in health care, (2) the accessibility of EHRs is an advantage, and (3) the possibility of giving health professionals a heads-up for any sensitivity or allergies to medicine is an important and positive aspect of the use of IT in health care.

#### Opinions on an Algorithm-Based Screening of EHRs

Out of the 5 respondents, 2 (P-1 and P-3) were exclusively positive about screening for both an increased risk or disposition for disease and inexpedient alcohol habits. One of the 5 (P-2) was predominantly positive but raised some concerns, while the remaining 2 (P-4 and P-5) were ambivalent, with no clear preference given to neither the positive nor negative aspects they mentioned.

The 2 patients who were exclusively positive about screenings said that they thought the screenings would be a beneficial tool for preventive care and, ultimately, a help for the patients. As for the respondent (P-2) who was predominantly positive, but had raised some concerns, these concerns were primarily linked to the screening of inexpedient alcohol habits; he was concerned that the knowledge following this kind of screening could be the subject of abuse, but did not elaborate on what kind of abuse. Regarding the screening for increased risk for disease, he did not express the same type of concerns.

The 2 patients who were ambivalent said they were positive about the screenings as a useful tool for preventive care. Yet, at the same time, they were somewhat skeptical about the potential of misusing both the information about increased risk of certain diseases and the information about inexpedient alcohol habits by, for example, insurance companies. Another major concern expressed by P-4 was the risk of false positives and how this could affect a patient’s life. Having experienced the consequences of a false positive herself, she was naturally concerned about this aspect of screenings. P-5 expressed concerns about how the results of screenings can have negative effects, such as stigmatization of the patient, which could result in inferior treatment. Although both P-4 and P-5 could see the benefits of these screenings, it was not entirely clear whether the positive aspects could outweigh the negative ones. In other words, in their answers to these questions, it is not clear whether they were predominantly positive or negative about screenings. Therefore, they were categorized as ambivalent. However, as shown in the next section, they might lean a little toward the more positive side.

When asked about whether they would personally agree to let their EHRs be screened for inexpedient alcohol habits, 3 of the patients (P-1, P-2, and P-3) said yes, unconditionally, while the remaining 2 (P-4 and P-5) were predominantly positive but had some reservations on the matter. Two of those who were exclusively positive about letting their personal EHRs be screened for inexpedient alcohol habits were also positive about screening in general. P-2, who expressed concerns about screening in general, did not express the same concerns about having his personal EHRs screened.

P-4 and P-5, who were ambivalent about screening in general, expressed a more positive attitude about letting their personal EHR screen for inexpedient alcohol habits. P-4 stated that her positive attitude was probably conditioned by the fact that she knows that alcohol is not a problem for her. If it was, she would likely feel less comfortable with the screening but would still allow it because she would like to know the result. P-5’s reservations were not about the screening itself but rather about the potential misuse of the information that the screening can produce. It was very important for her that the results or information from the screening would only be used in a way that helps and benefits the patient and not in any negative way (eg, condemnation and stigmatization). Although both could see personal advantages and, therefore, would like to know the results of a screening of their personal EHRs, they also expressed reservations about the matter. That they did not altogether reject the idea of having their personal EHRs screened for inexpedient alcohol habits is an indication that they were leaning toward a more positive attitude about screening in general.

#### Being Asked Based on a Reminder

The last aspect of the overall question about patients’ attitudes and opinions about an algorithm-based helping tool was how they would feel about being *asked* about this subject based on a screening. Three out of the 5 patients (P-1, P-2, and P-3) had no reservations about being asked based on a screening and said that they would be comfortable with this. The remaining 2 respondents (P-4 and P-5) had some reservations about the matter; these reservations did not concern the screening per se, but rather about *how* the health professionals would handle the asking, based on being reminded. For them to be comfortable about being asked would depend on *the way* the health professional asked them. However, the importance placed on how they are asked about alcohol habits was important for them, *regardless* of the reasons behind them being asked. In other words, being comfortable about being asked about alcohol habits depended on *how* they were asked. Further, they mentioned that they would like to be informed about the reasons behind being asked—whether the reason is a screening, routine, or suspicion. This means that the reservation they had about being asked was not linked to the screening per se, but to being asked in general.

## Discussion

### Summary

As a preparatory step before the development of an algorithm and a subsequent helping tool that would present notifications in the EHR by means of algorithms screening the data if the patient was considered to have a possible harmful use of alcohol, we conducted interviews to investigate the attitudes and ethical considerations among patients and health professionals at hospitals toward such a helping tool. The health professionals had both positive and negative opinions about such an algorithm-based helping tool. On the positive side, the health professionals noted that the helping tool would save them some much needed time by providing a quick overview of the information in the EHR and ensuring important tasks such as addressing harmful use of alcohol would not be forgotten. However, this positive attitude was conditioned by a number of requirements: That the tool would not be time-consuming and would be adapted according to relevance and usefulness, that it would work and be user-friendly, and that sufficient resources would be spent on implementation. On the negative side, the health professionals noted concerns that this type of helping tool would take away the health professionals’ instinct, because they might follow recommendations blindly without exercising professional discretion, and they also have concerns of a more fundamental nature, questioning the algorithm’s capability of making and giving clinical decisions and recommendations. The patients were overall very positive about the idea of an algorithm-based screening tool, saying that they would consider such a tool beneficial for preventive care, thereby ultimately helping patients in need for advice about alcohol habits. However, some expressed concerns that such a tool would provide information that could be misused, that the screenings could result in stigmatization and inferior treatment, and that false positives could impact patients’ lives.

### Comment on the Practical Nature of the Health Professionals’ Answers

Something very characteristic about the health professionals’ positive and negative opinions about both the general use of IT in health care and the algorithm-based helping tool was that—with a few exceptions—they were very practical. In other words, their justifications for being both positive and negative were related to how IT and the helping tool would affect them in a practical way. Only 2 health professionals expressed opinions about how the use of IT and, specifically, an algorithm-based helping tool might affect the nature of health care in a more general way. Even though we did not ask them specifically about this more general and ethical perspective, it is interesting that so few brought it up, and that they only gave practical justifications. There can be a number of reasons for this: (1) we did not ask them specifically about a more general perspective on the use of IT in health care, (2) they did not have any reflections on the more general and ethical perspective, or (3) the time frame for the interview was short, and they were at work. However, a focus on the practical perspectives could also be a useful and an important insight for anyone developing and implementing these systems. Indeed, if you want health professionals to use and comply with a new IT system, these professionals must be included in the development and implementation processes, that is, in how the systems will affect them in a practical way.

### Ethical Issues

The introduction and use of algorithms can essentially change the health care system and how medicine is practiced today. Therefore, it is important to take seriously how the use of algorithms can ethically challenge the fundamental aspects of medical practice. Some of the ethical challenges that might arise out of using algorithm-based helping tools are (1) the patient’s privacy, (2) the autonomy of health professionals, and (3) the relationship between the patient and health professional.

Using algorithm-based helping tools to screen previously collected health data has obvious gains regarding patient beneficence. At the same time, however, this screening can be a possible breach of privacy because of the flow of data from one context to the other, as data collected in one context may be used for an algorithm-based screening in another context. These screenings can be a useful tool in the preventive treatment of, for example, inexpedient alcohol habits. However, the ethical tension between beneficence and privacy arises because the screening may give health professionals access to information about the patient that he/she has chosen not to disclose or found irrelevant or inappropriate in that specific health care context. Therefore, it is important to weigh the concern for patient beneficence and patient privacy when developing and implementing this type of algorithm-based helping tool.

A central aspect of professional autonomy is the exercise and cultivation of *professional practical wisdom* [22]. Such practical knowledge entails that the professional considers a broad range of possible issues, decisions, and actions when contemplating clinical decisions. Another way of putting this is to say that professional practical wisdom makes good, professional discretion possible. In the context of professional autonomy, it is useful to distinguish between algorithm-based helping tools that give clinical decision *support* via recommendations, and tools that *make* clinical decisions because they have different impacts on professional autonomy, with the latter being an authoritative helping tool. If the tool is used to offer *support,* this can be very constructive for the exercise and cultivation of professional practical wisdom because it can draw attention to important considerations that the professional might not otherwise have given thought to. If the tool is used to *make* clinical decisions, it can become a threat to professional autonomy because it restricts the practitioner’s ability to exercise professional discretion. However, constitutional constraints, such as time pressure and the design of the helping tool, can influence whether the clinical support tool is used and perceived as an authoritative tool or not, if, for example, because of time pressure or because the recommendation is phrased in an authoritative way, the professional follows the recommendations blindly without exercising professional discretion. This would, de facto, mean that a clinical decision *supportive* tool would become a clinical decision-*making* tool, thereby posing a threat to professional autonomy, because a clinical decision-making tool leaves no room for exercising professional practical wisdom.

The patient-centered relationship is currently the most widely accepted ideal for the doctor–patient relationship in the Western world [23]. This type of doctor–patient relationship is characterized by the following 5 aspects: (1) the biopsychosocial perspective, (2) the patient-as-person, (3) shared power and responsibility, (4) the therapeutic alliance, and (5) the practitioner-as-person [24]. Introducing and using algorithm-based helping tools can have an impact on 4 out of the 5 central aspects of the patient-centered relationship, leaving only the fifth aspect, the practitioner-as-person, untouched. One of the aspects that could be affected is the shared power and responsibility between the doctor and patient; this aspect entails that the doctor and patient are equal in their autonomy and authority because they both possess expert knowledge—the patient about personal needs and preferences, while the doctor has the required medical knowledge—which is essential to the shared decision-making process. The patient’s autonomy in the patient-centered relationship is based on being *heard* and *receiving* expert medical knowledge, making an informed decision possible. By contrast, the doctor’s autonomy, in this aspect, is based on *having* medical knowledge. Algorithm-based helping tools can interfere in this central exchange of knowledge if the doctor does not understand the decisions or suggestions of the helping tool because of, for example, black boxing, thereby restricting both the patient’s and doctor’s autonomy by not being able to respectfully receiving and giving expert knowledge. Introducing and using algorithm-based helping tools can intervene in the doctor–patient relationship in 4 out of these 5 central aspects of the patient-centered relationship and, ultimately, change this relationship into a more paternalistic relationship, where the autonomy and authority are centered around algorithms, not the doctor and patient.

### Limitations

This study has 3 main limitations: (1) the small sample size, (2) the patient’s sometimes restricted ability to participate in the interviews, and (3) the physical frameworks of the interviews. Even though the correlation among respondent answers was good, this is a limitation. An obvious way of furthering this study would be to make a quantitative investigation based on the same research questions. This would ensure a larger population, thereby strengthening the study. The second limitation relates to the admitted patients who did not necessarily have sufficient energy and strength to participate in an in-depth interview. Even though we ensured that the health professionals approved of the patients’ participation, this is not necessarily a guarantee. This limitation was obvious when we interviewed the nonadmitted patients, who had significantly more mental surplus and, therefore, gave more nuanced and lengthy answers. The third limitation concerns the physical frameworks of the interview. Some of the admitted patients were admitted in multibed wards and were, therefore, interviewed with other patients present. This may have influenced their level of comfort in being interviewed and, ultimately, their answers. The interviews with health professionals were conducted in a small office away from patients. Even though the interviews were held at a distance from their respective departments, they nevertheless were at work and, therefore, had limited time available. Hence, the situation they were in right before the interview—which might have been a difficult one—could have influenced their concentration and mental presence.

### Recommendations for Development and Implementation

This study’s results highlight the importance of designing algorithmic helping tools to be as transparent as possible. Another way of putting this is to say that the helping tool must not be designed to only provide yes/no answers. This would, de facto, “black box” important information and make the health professionals unable to use professional discretion to evaluate the recommendation made by the helping tool. Therefore, an algorithmic helping tool should provide some insight into why a recommendation was given. This way, the health professional will be allowed to judge whether to agree or disagree with the recommendation based on the more detailed information.

Another key result highlights the importance of including health professionals in the development and design process of algorithmic helping tools; indeed, they hold important and valuable knowledge about what key factors will increase use of such a system. This is important to ensure that the helping tools being implemented are, indeed, helping health professionals and not creating frustration in an already busy work environment.

## Abbreviations

EHR: electronic health record
IT: information technology
SBIRT: screening, brief intervention, and referral to specialized treatment
